# Extended trochanteric osteotomy in revision hip arthroplasty: a case series study and systematic literature review

**DOI:** 10.1186/s42836-022-00115-w

**Published:** 2022-04-03

**Authors:** Khalid Hamad, Sujith Konan

**Affiliations:** 1st Floor, Orthopaedic department, UCLH, 250 Euston Road, London, NW1 2PG England

**Keywords:** ETO, Extended trochanteric osteotomy, Revision hip arthroplasty, ETO union rate, Aseptic loosening, Prosthetic joint infection

## Abstract

**Background:**

Extended trochanteric osteotomy (ETO) in revision hip arthroplasty provides direct access to the femoral medullary canal and facilitates removal of implants and re-implantation. This study looks at objective outcomes of ETO from a systematic review of the literature and a case series of revision total hip arthroplasty (THA) cases with ETOs from the authors’ local institution.

**Methods:**

(1) The National Institutes of Health (NIH) national library of medicine was searched for studies related to ETO and the preferred reporting items for systematic reviews and meta-analyses (PRISMA) technique were followed.

(2) Case series of 23 revision THAs with ETOs from University College London Hospital (UCLH) were retrospectively analyzed with a minimum of 2-year follow-up for radiological outcomes.

**Results:**

(1) The main revision THAs diagnoses were aseptic loosening (880/1,386; 63.4%), prosthetic joint infection (PJI) (301/1,386; 21.7%) and periprosthetic THA fractures (78/1,386; 5.6%). Other diagnoses, including non-specified reasons for THA revision in the chosen studies, accounted for 9.2% (127/1,386). The total mean was a union rate of 95.2%, an infection eradication rate of 91.6%, a femoral stem subsidence rate of 16.6%, with the rate of subsidence more than 5 mm being 10.7%. ETO proximal migration was reported in 7.8% of ETOs; however, it rarely required re-attachment (0.9%). Intraoperative fracture during revision THA with ETO was reported to be at a rate of 5%; while postoperative femoral fracture rate was at 7.8%.

(2) All 24 cases had radiographic union at 3 to 6 months and there was no reported femoral stem subsidence.

**Conclusion:**

The overall outcome of this literature review provides moderate-quality evidence indicating that ETO provides safe outcome for revision THAs in single and 2-stage revision surgeries with low ETO non-union, femoral stem subsidence, greater trochanter (GT) proximal migration and fracture rates in the different diagnoses groups of revision THA at over 2-year follow up. In the case series group, there was radiographic union of all ETOs with no reported femoral stem subsidence or periprosthetic fractures.

## Introduction

Adequate surgical exposure is essential in successful revision hip surgery and ETO can be a useful tool in achieving that for the appropriate indications. An ETO in revision THA can be used to provide access to the femoral implant removal, enhance exposure of the acetabulum and correct any varus deformity in un-cemented femoral revision. This has the added advantage of keeping the soft tissue proximally and distally uncompromised [1].

Revision of the femoral stem is indicated in failed primary hip replacement, single stage or 2-stage revision of infected hip replacement, and in revision of failed proximal femoral fracture fixation. Revision with posterior approach is often preferable due to its extensile exposure even with a previous lateral approach. This avoids further damage to the gluteus medius and minimus, and in revision of failed femur fixation it allows full exposure and hip dislocation prior to removal of metal work [2].

Younger described series of Paprosky’s extended proximal femoral osteotomies (EPFO), later named ETO, and advocated its use in revision surgery. EPFO technique was described for removal of distally fixed femoral components. The anterolateral proximal femur is cut on one third of its circumference, extended distally and levered on the anterolateral hinge of the periosteum and muscle, creating an intact muscle osseous sleeve. ETO is composed of greater trochanter and anterolateral femoral diaphysis along with preserved gluteus medius and vastus lateralis attachments as described by Younger and Paprosky [3]. It was performed via the posterolateral approach. The surgical equipment described in the preoperative planning schedule includes explant tools, trephines, high speed burrs, flexible osteotomes, and ultrasonic cement removal instruments to aid cemented stem removal. Finally, the ETO can be made after dislocation and stem removal in infection cases for thorough debridement of the canal or in loose femoral stem cases with proximal varus remodelling for better access to distal femur [4].

The anterolateral aspect of the proximal femur is cut with an oscillating saw, and then multiple pencil burr drill holes on the osteotomy lines are connected using an osteotome. The anterior soft tissue is left undisturbed with this controlled fracture through the perforated holes.

When using the technique of cemented impaction, allograft at the distal femoral osteotomy site with cancellous bone is associated with high union rate at a mean of 6 months. Care must be taken to ensure that extrusion of cement does not occur to allow unimpaired bone healing in cemented polished prosthesis at revision THA with ETO [5].

Postoperatively, femoral revision may be treated with protected partial weight bearing (30% weight bearing) for the first 6 weeks. After 6–8 weeks, patients can weight-bear as tolerated, but typically avoid active abduction for 6–12 weeks until radiographic union of the osteotomy [6].

Revision THA with ETO is associated with lower stem subsidence rate and less cortical perforation compared to revision THA without ETO [7].

We reviewed our minimum 2-year results of ETO for femoral revision focusing on radiological and clinical outcomes.

We also conducted a systematic review to look at the objective outcomes of ETO, including union rate, infection eradication rate, subsidence and proximal GT migration in the setting of revision THA for prosthetic joint infections, aseptic loosening and periprosthetic fractures. To our knowledge, this is the first systematic review to look at the outcomes in all the three categories mentioned.

## Methods for cases series

The senior authors’ database of revisions was reviewed and total number of revisions where ETO was used was identified. Clinical indications and clinical and radiological outcomes at minimum two years were identified. A total of 83 revisions were identified on a minimum 2-year follow-up. Twenty-four cases required ETO. The average age group was 72 years (range, 65–91). There were 11 females and 13 males. The indications for ETO were removal of distal cement in 10 cases, removal of well-fixed femoral component in 7 cases, periprosthetic fractures in 4 cases and revision for varus remodelling in 3 cases.

### Method and data collection for systematic review

The letters “ETO” and the words “Extended Trochanteric Osteotomy” were searched in PubMed from 1990 to date separately and 132 studies were retrieved (note: ETO was first described in 1995 by Younger et al. [3]). 18 studies were not relevant to the topic of this research; hence they were omitted. The remaining studies were reviewed individually for relevance to outcomes of trochanteric osteotomies and 94 studies were excluded. Against the inclusion criteria, 20 studies were finally included in this study.

#### Inclusion criteria

Studies published in English describing outcomes of ETO in revision THA with radiographic data and follow-up for at least 1 year and more than 5 patients included.

#### Exclusion criteria

Revision THAs with surgical approaches other than posterolateral approach, modified ETO technique, systematic reviews, and studies that failed to fulfil the inclusion criteria.

The search and the final inclusion of studies were carried out by the first author and the resultant studies were subsequently reviewed and affirmed by a senior author. All the 20 papers included were level IV and III retrospective studies. The total number of cases was 1,386 and they all received revision THA using standard ETO through posterior/posterolateral approach. No modified ETOs were included and hence no other surgical approaches were included in this study. No primary THAs, regardless of complexity, were included either in this study.

The main revision THAs diagnoses were aseptic loosening (880/1,386; 63.4%), prosthetic joint infection (PJI) (301/1,386; 21.7%) and periprosthetic THA fractures (78/1,386; 5.6%). Other diagnoses including non-specified reasons for THA revision in the chosen studies accounted for 9.2% (127/1,386).

## Results of case series

ETO was successfully performed in all 24 cases. There were no immediate complications of fractures or soft tissue damage. All revisions with ETO were completed with monobloc revision titanium systems. Radiological union was confirmed between 3–6 months in all cases (100% union rate). Thigh pain was noted in 14 cases (58.3%) at a 6-week follow-up and the pain resolved by 6 months. No stem subsidence was observed in any case. All patients commenced weight bearing with crutches and ETO did not change their mobility postoperatively.

### Results of systematic review

#### Radiographic union

The total mean union rate was 95.2% (1,319/1,386). The average ETO union rate for aseptic loosening was 95.02% (836/880) while the average rate for PJI ETO union was 96.8% (291/301) at an average 2-year follow-up from the 2^nd^ stage THA revision; ETO for periprosthetic fractures Vancouver B2/3 was 98.6% (77/78) at 2 years follow up on average. Other and non-specified reasons for revision THA had an average ETO union rate of 82% (104/127) at an average 1-year follow-up.

#### Infection eradication rate

Four studies [8–11] reported data on infection eradication rate, with the average eradication rate being 91.6% (98/107). Standard cemented femoral stem was associated with fewer complications, including infection, fracture and dislocation in revision surgery compared with cemented long femoral stems. Additionally, standard stem could preserve the distal bone stock [12].

#### Femoral stem subsidence

In three studies [12–14] data were detailed on femoral stem subsidence measured in millimetres. The total average rate of femoral stem subsidence in ETO THA reported in these studies was 16.6% (14/84). Only 10.7% (9/84) of femoral stems underwent subsidence of more than 5 mm.

ETO proximal migration was reported in 7.8% (55/702) [7, 12, 15–19] of the subjects, and it rarely required re-attachment operation (0.9%; 1/108) [20]. Intraoperative fracture during revision THA with ETO was reported in 5% (32/640) while postoperative femoral fracture was reported in 7.8% (45/574) [7, 13, 21–23] of the subjects. 12.5% of them needed reoperation for different reasons, including non-union, subsidence, GT reattachment and infection [7, 18, 20–22]. However, the majority of reoperations were not for removal of acetabular or femoral components. The dislocation risk was 4.7% following revision THA with ETO (2/42) [13, 16].

Modular fluted tapered distally femoral stem had a union rate of 90.2% (92/102) [8, 16]. Cortical strut allograft can be useful in Paprosky grade IIIA and higher to address bone loss. However, its use in lower grade bone loss is questionable as it is associated with lower union rate. Porous coated uncemented femoral stem has a high ETO union rate of 99.1% (116/117) [20, 22]. Other studies reported that the combination of uncemented femoral stem had a union rate of 95.5% (64/67) [18, 24]. ETO non-union rate with the uncemented revision THA was 3 times lower than that with the cemented revision THA [7].

## Discussion

ETO is useful in THR infection treatment in two-stage revision surgery where femoral components could not be extracted with standard techniques. It is associated with a  infection resolution rate of 87%, and union rate reported to be at 95% at 11.5 weeks [19] and at 98% at 4 months. Superior GT migration less than 5 mm was reported in 7% of ETOs, while early loosening was reported to be at 1.3% at 4 months after operation. Complications also included osteotomy fragment fracture in 5.4% [19].

While in aseptic single THA revision, overall union rate was reported to be at 93.1%, and the rate of femoral stem subsidence > 5 mm was 7.1%, the results were similar between THA revisions with ETO for periprosthetic fractures and THA revisions with ETO for reasons other than fracture. Older age and prior femoral cementation may be negative factors in ETO union rates [23]. Persistent pain and painful hardware can also occur as postoperative complications; other complications include posterior dislocation, traumatic periprosthetic fracture [25].

Seung-Jae Lim et al., in 2011, compared a cohort of non-infected patients who received revision hip arthroplasty using ETO with a periprosthetic hip infection group. The hip infection group had infection eradication rate of 96%, a union rate of 100% at 10.6 weeks, rate of proximal migration > 2 mm of 8%, a rate of intraoperative femoral crack of 13%, a rate of femoral stem subsidence > 5 mm of 4%, a periprosthetic fracture rate of 8%, and a dislocation rate of 4%. The non-infection group reported a 96% union rate at an average of 10.4 weeks, a rate of proximal migration > 2 mm of 8.6%, an intraoperative femoral crack rate of 10.8%, a rate of subsidence > 5 mm of 8.6%, a periprosthetic fracture rate of 4%, and a dislocation rate of 2% [13].

Similar findings were observed in a study of a mainly single-stage cementless ETO revision hip arthroplasty involving 166 patients, with a union rate of 98.8% at 2-year follow-up. In the study, 71% united within 3 months, 0.6% had malunion, 1.2% had proximal segment migration > 2 mm, 2.4% had fracture of osteotomy fragments, 92.1% had bony ingrowth 2-years after operation, 10.2% had dislocation and 10.2% received a re-operation [24].

When using fluted and tapered modular distal femur fixation stem with or without extended trochanteric osteotomy, the rates of cortical perforation and marked stem subsidence > 5 mm were significantly higher in the group treated without an ETO. However, when stratified in terms of bone defect, no significant difference was found [10].

Delayed extended trochanteric osteotomy fixation in two-stage cemented arthroplasty with interval placement of antibiotics-impregnated cement spacer is associated with a high union (up to 100%) rate at 6 months, and a subsidence rate of 15%. Delayed ETO fixation with interval placement of articulating antibiotic-impregnated cement spacer permits reliable healing of the osteotomy [12].

Antibiotic-coated prostheses (ACP), such as Zimmer-Biomet Stage One Select Femoral Spacer (ZBSO) used in stage-1 revision surgery in the setting of ETO like moulded or hand-made spacers have high rate of spacer fracture, with ACP spacer fracture rate being at 25% due to its thin core, compared to a rate of 2.4% without ETO. This implant should be used with caution with an ETO, and an alternative ACP should be considered when performing ETO [26].

A comparison between ETO length (average at 14.7 cm), and modified sliding trochanteric osteotomy length (average at 6.1 cm) has shown that the length of osteotomy is negatively correlated to the GT migration distance [27]. Hence, osteotomies shorter than 10 cm are at high risk of developing over 1 cm GT proximal migration, especially without adequate distal cerclage wire fixation [5]. Sliding trochanteric osteotomy has a non-union rate 4 times the non-union rate observed in ETO cases, [28] at least partly due to its decreased length.

Cortical strut allograft with ETOs can be used in cases where bone stock restoration is needed with no significant difference in functional outcome, subsidence, alignment or migration. There is an insignificant lower union rate with the use of cortical strut allograft. In mild to moderate cases of bone stock loss (Paprosky grade I & II), excellent clinical outcome and high union rate were reported in ETOs without strut augmentation [29].

The use of cerclage wire with ETO does not seem to be related to reduced infection eradication rate or reduced osteotomy union in the first stage of a two-stage revision surgery for chronic infection [11].

Fixation of the ETO using 3 or 2 cables showed no significant difference in stiffness, peak force, or displacement in biomechanical model [30].

At 1 year follow-up, there was some evidence that union rate of ETOs was higher with trochanteric metallic reinforcement plates (MRP plate) compared to cables and metallic wires [31].

The main limitation of the case series study is that it mainly relied on radiographic measures and had limited functional outcome assessment. Another limitation from the literature review is that there was evident disparity in the follow-up periods, ranging between 1 year to several years, while this lack of uniformity did not generally affect the shorter-term outcomes, it can affect some long-term conclusions derived from these results.

## Conclusion

Overall, this literature review provides moderate-quality evidence that ETO yielded safe and effective outcome for revision THAs in single- and two-stage revision surgeries with low rates of ETO non-union, femoral stem subsidence, GT proximal migration and fracturein revision THA for different reasons at an over 2-year follow-up.

Revision THA for the three main reasons showed excellent results with ETO, with a radiographic union rate of 95% for aseptic loosening, 96.8% for prosthetic joint infection following two-stage revision surgery (with an infection eradication rate of 91.6%) and 98.6% for revision THA for periprosthetic fractures. All three groups showed low complication rate, a rate of 10.7% with femoral stem subsidence of over 5 mm and a rate of 7.8% with ETO proximal migration.

In this case series group, there was radiographic union of all ETOs with no reported femoral stem subsidence or periprosthetic fractures. This provides evidence that supports the effective and safe use of ETO in removal of distal cement, removal of well-fixed femoral component, varus femoral remodelling and periprosthetic fractures in older patients.

## Data Availability

Supporting data of the manuscript are available on reasonable request.

## Abbreviations

ETO: Extended trochanteric osteotomy
THA: Total hip arthroplasty
NIH: National Institutes of Health
PRISMA: Preferred reporting items for systematic reviews and meta-analyses
UCLH: University College London Hospital
GT: Greater trochanter
EPFO: Extended proximal femoral osteotomy
PJI: Prosthetic joint infection
ACP: Antibiotic-coated prostheses
ZBSO: Zimmer-Biomet stage one
MRP: Metallic reinforcement plate
